# Hydrogen-Bonded Organic–Inorganic Hybrid Based on Hexachloroplatinate and Nitrogen Heterocyclic Cations: Their Synthesis, Characterization, Crystal Structures, and Antitumor Activities In Vitro

**DOI:** 10.3390/molecules23061397

**Published:** 2018-06-08

**Authors:** Jin Zhao, Fuming Chen, Yutong Han, Huaqing Chen, Zhidong Luo, Hao Tian, Yi Zhao, Aiqing Ma, Longguan Zhu

**Affiliations:** 1 School of Pharmacy, Guangdong Medical University, Dongguan 523808, China; vincent-vg@163.com (J.Z.); fm.chen@siat.ac.cn (F.C.); hyt7570@163.com (Y.H.); luozhidong06@126.com (Z.L.); tianhao5588@126.com (H.T.); zhaoyicomnet@gdmu.edu.cn (Y.Z.); 2 Guangdong Key Laboratory of Nanomedicine, CAS Key Lab for Health Informatics, Shenzhen Engineering Laboratory of Nanomedicine and Nanoformulation, Shenzhen Institutes of Advanced Technology (SIAT), Chinese Academy of Sciences, Shenzhen 518055, China; chenhuaqing99@163.com; 3 Department of Chemistry, Zhejiang University, Hangzhou 310027, China

**Keywords:** organic–inorganic hybrid complexes, structure, hydrogen bonding, antitumor activities

## Abstract

Three new crystal structures containing [PtCl_6_]^2−^, pyridinium and benzimidazole groups have been prepared: [PtCl_6_]·(H-bzm)_2_·2(H_2_O) (**1**), [PtCl_6_]·(H-bipy)_2_·2(H_2_O) (**2**), [PtCl_6_]·(H-dimethyl-bipy)_2_·2(H_2_O) (**3**) [H-bzm: benzimidazole cation, H-bipy: 2,2′-bipyridine cation, H-dimethyl-bipy: 4,4′-bimethyl-2,2′-bipyridine cation]. All compounds have been fully characterized by elemental analyses, single-crystal X-ray analyses, IR spectra, TG analyses, and fluorescence studies. Single-crystal X-ray diffraction analysis suggests that the primary synthon contains ^+^N–H···Cl^−^, including ionic bonding and hydrogen bonding interactions. The dimensions are enhanced further by secondary O–H ∙∙Cl and N–H ∙∙O hydrogen bonding interactions between donor and acceptor atoms located at the periphery of these synthons. Moreover, coulombic attractions between the ions play an important role in reinforcing the structures of these complexes. In addition, antitumor activity against human lung adenocarcinoma cell line (A549) and human nasopharyngeal carcinoma cell line (CNE-2) was performed. These complexes all showed inhibition to the two cell lines, while complex **3** exhibited higher efficiency than complexes **1**–**2**.

## 1. Introduction

Due to its wide range of applications in materials science, molecular recognition, the pharmaceutical chemistry, and so on [1,2,3], crystal engineering has attracted interest from chemists, medical researchers, and materials scientists in the past few decades. Most work has focused on the controlled formation of supramolecular networks, not only by covalent bonds and coordinate covalent bonds, but also by the use of noncovalent interactions such as electrostatic interactions, hydrogen bonding, and *π*–*π* stacking interactions [4,5,6]. It is clear that hydrogen bonding plays a crucial role in controlling the molecular arrangement and further properties of crystal materials due to its moderate strength, directionality, and flexibility [7,8]. Moreover, the molecular packing in crystals is an optimum balance of different intermolecular interactions. Hence, even a small change of building blocks can result in quite distinct hydrogen-bonding structures and further crystal packing [9].

**Scheme 1:**
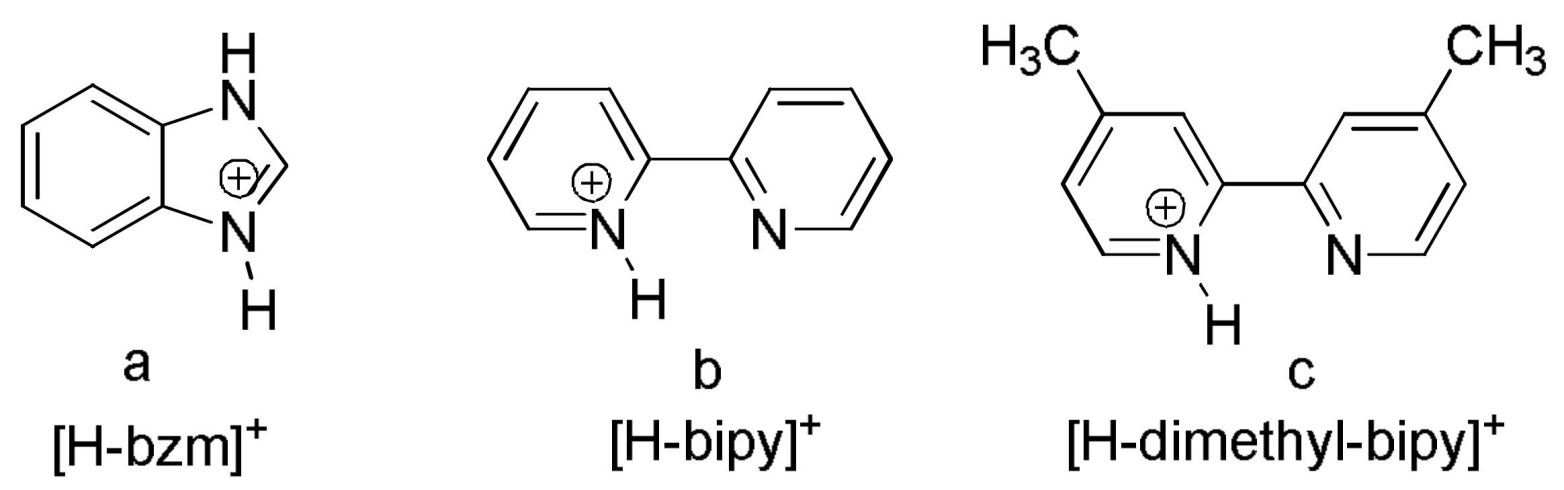
Organic cations to form the target complexes.

Recently, angular pyridinium cations and their derivatives have been successfully employed to construct a series of molecular cocrystals with a variety of organic synthons or metal complexes such as perhalometalates [10,11,12], since the cations are the hydrogen-bond donors, linking the anionic ions that act as hydrogen-bond acceptors through coordinative bonds and/or N–H ∙∙Cl–M interactions to enhance structural diversity [13,14]. And a series of crystal structures containing pyridinium cations and potassium tetrachloroplatinate have been cocrystallized with the hydrogen bond donor groups (N–H) through ^+^N–H…Cl^-^ interactions [15,16,17]. The use of ionic building blocks enhances the hydrogen bonding energy and thus the crystal lattice energy, which increases the robust mess of the synthons, displaying diverse extended hydrogen-bonded networks from 1D to 3D [11,18,19,20]. Based on the above points, we have chosen hexachloroplatinate as hydrogen-bond acceptor to build M–Cl…H–N hydrogen bonds. Also, the protonated bipyridine (Hbipy)^+^ and benzimidazole (Hbzm)^+^ were chosen to enrich hydrogen-bonded crystalline systems, giving rise to different molecular conformation and further crystal packing modes. Here, three complexes were reported, which have been prepared by reacting potassium hexachloroplatinate (K_2_PtCl_6_) with the benzimidazole cation, bipyridinum cation and its relatives (Scheme 1). With the participation of solvent water molecules in the cocrystallization, the three complexes, [PtCl_6_]·(H-bzm)_2_·2(H_2_O) (**1**), [PtCl_6_]·(H-bipy)_2_·2(H_2_O) (**2**), [PtCl_6_]·(H-bimethyl-bipy)_2_·2(H_2_O) (**3**), are aggregated through N–H…Cl, O–H…Cl and N–H…O hydrogen-bonding interactions, with coulombic attractions between the cations and anions, building up supramolecular structures. The crystallographic data and refinement parameters are in Table 1.

Moreover, Pt–organometallic compounds are among the most active and widely used clinical drugs in cancer chemotherapy [21,22,23]. For example, Marta groups have theoretically studied antitumor abilities of the complexes containing *N*-phenyl-guanidine derivatives and PtCl_3_^−^ and PtCl_2_ in different coordinating modes [24]. New platinum complexes are expected in the attempt to design novel chemotherapeutic agents [25,26,27]. Here, as new platinum complexes, the cytostatic activity of the three complexes against A549 and CNE-2 was screened by the MTT assay. All the complexes can inhibit proliferation of A549 and CNE-2 cells. Compared with **1**–**2**, complex **3** yielded the highest cytotoxicity. Further cell cycle analysis for complex **3** showed that it arrested both kinds of cells in G_0_/G_1_ phase.

## 2. Results and Discussion

### 2.1. Analysis of the Structures of ***1**–**3***

**Scheme 2:**
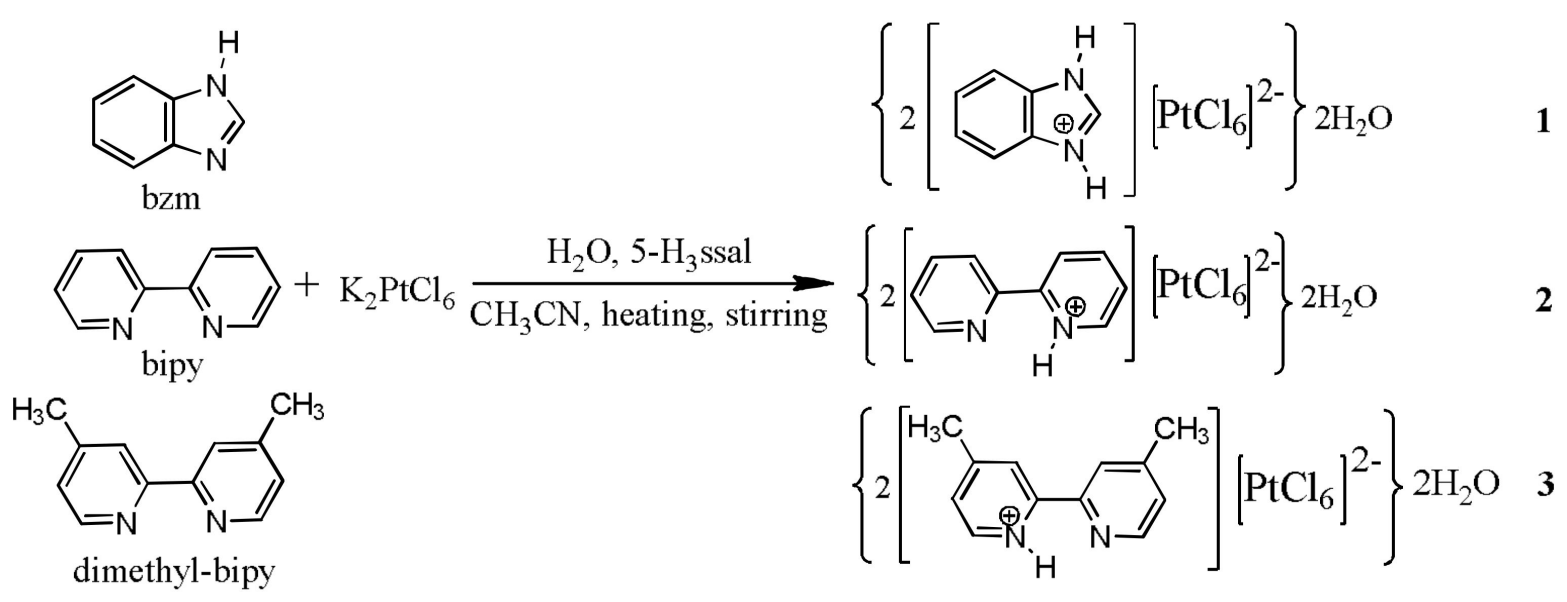
Synthesis route for complexes **1**–**3**.

**Figure 1:**
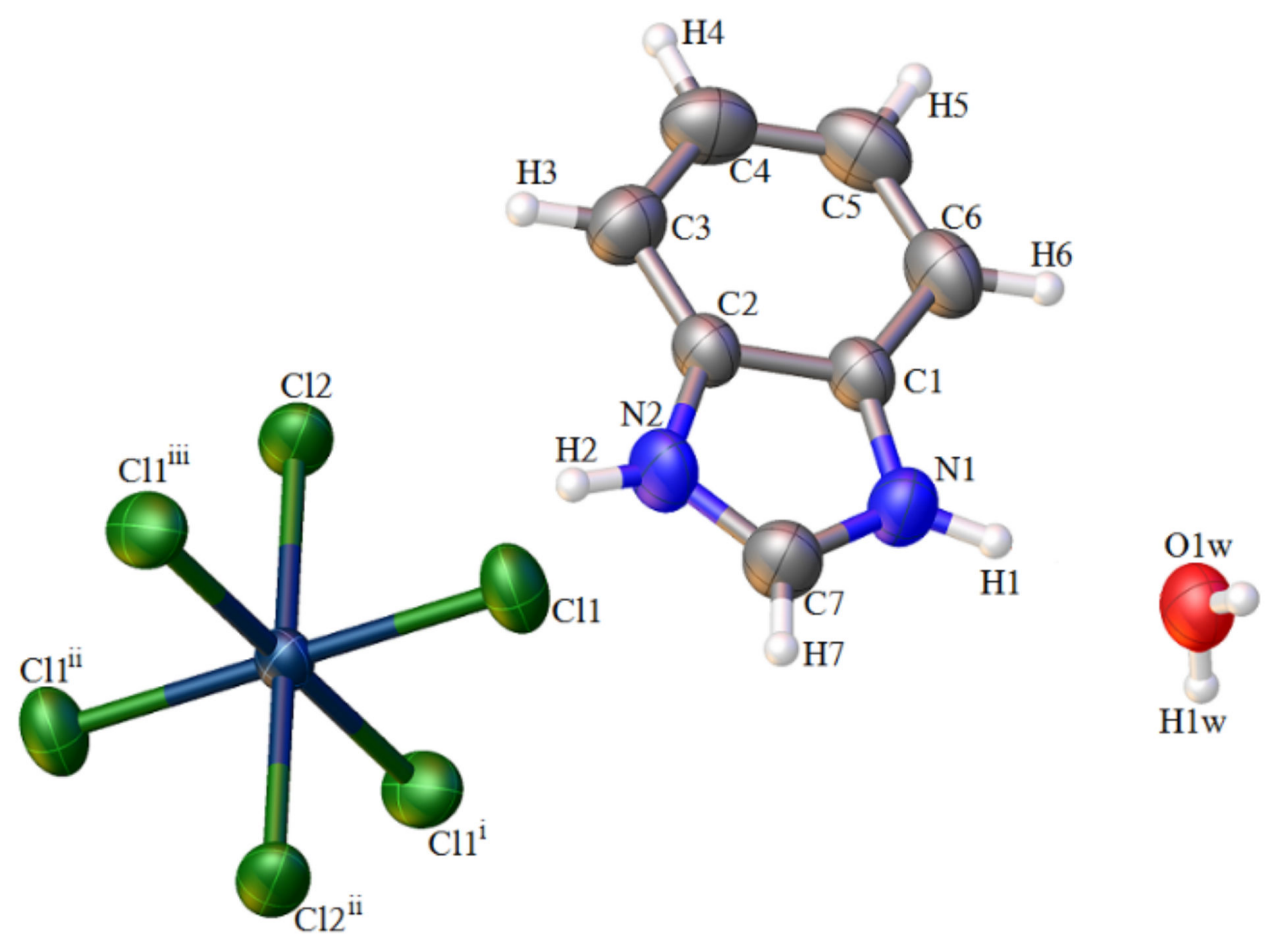
View of the asymmetric unit of complex **1** with the numbering scheme. The thermal probability is drawn at 30%. Symmetry codes: i = x, −y, −z; ii = −x, −y, −z; iii = −x, y, −z.

**Figure 2:**
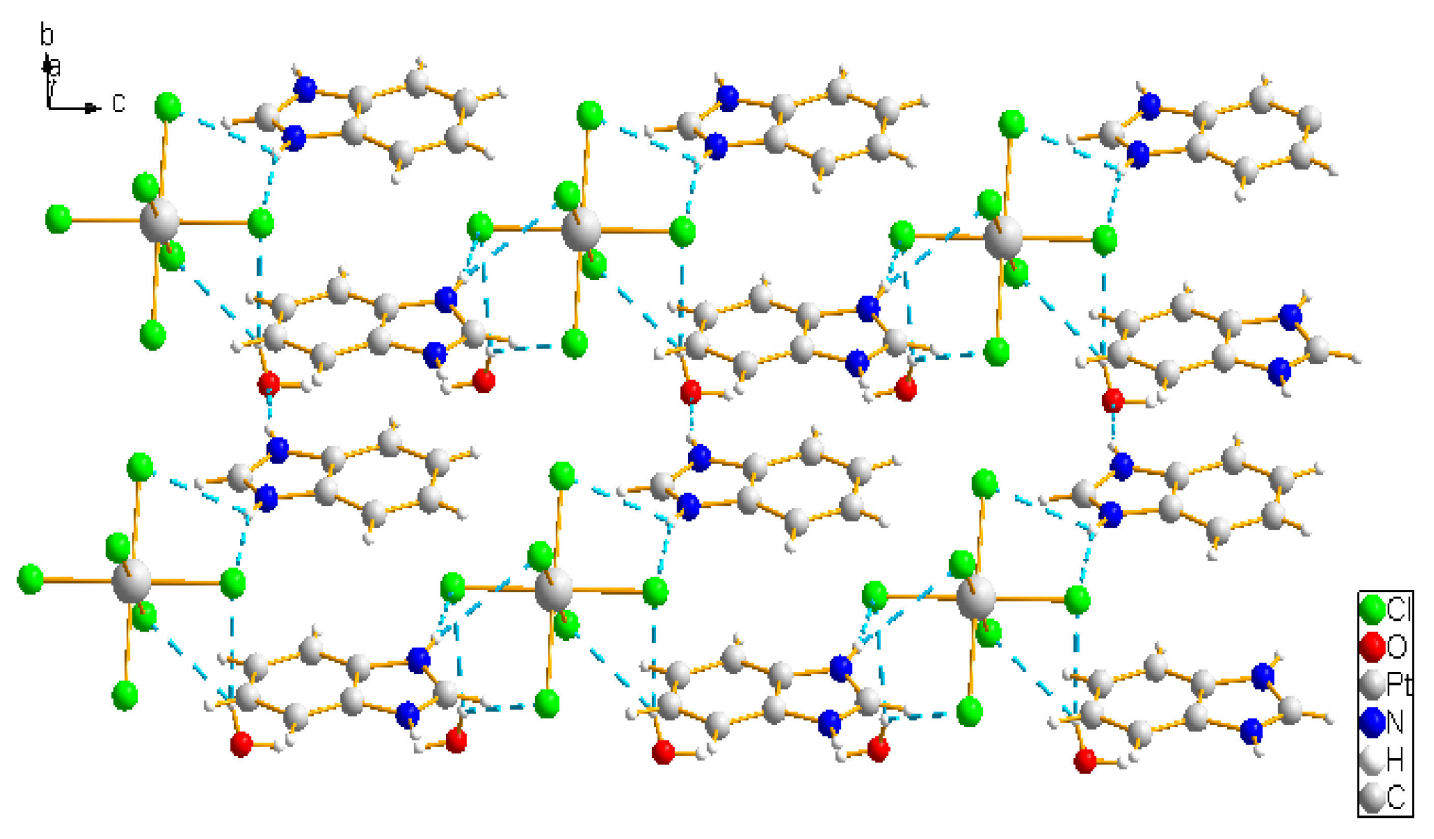
Hydrogen-bonded motifs for complex **1**. Hydrogen bonds are shown as dashed lines.

The three complexes were synthesized according to the routes in Scheme 2. 5-Sulfosalicylic acid dehydrate (H_3_ssal) acted as the donor of the proton H, transferring to the base N atom of benzimidazole, forming [H-bzm]^+^. As depicted in Figure 1, the structural determination of **1** reveals a three-component cation–anion species, in which each [PtCl_6_]^2−^ crystallizes with two benzotriazole monocation and two water molecules. Analysis of the crystal packing of **1** suggests that ^+^N1–H1 in [H-bzm]^+^ is bonded to the adjacent water solvent by hydrogen bond with the distance N1–H1 ∙∙O1w of 2.72 Å. O1w acts as a donor to form a weak hydrogen bond with two coordinated chloride ions in [PtCl_6_]^2−^ with the bonds O1w–H1w ∙∙Cl1 of 2.86 Å and O1w–H1w ∙∙Cl2 2.84 Å (Table 2). Moreover, weak intermolecular interaction is included between N–H ∙∙Cl with the distance of 2.51 Å, which is still in the range of the sum of van der Waals radii (2.54 Å). All these hydrogen bonds help to generate a 2D network (Figure 2). In addition, as [PtCl_6_]^2−^ is negative and [H-bzm]^+^ is positive, the coulombic attractions between the two ions play an important role in building up the crystal structure [28].

**Figure 3:**
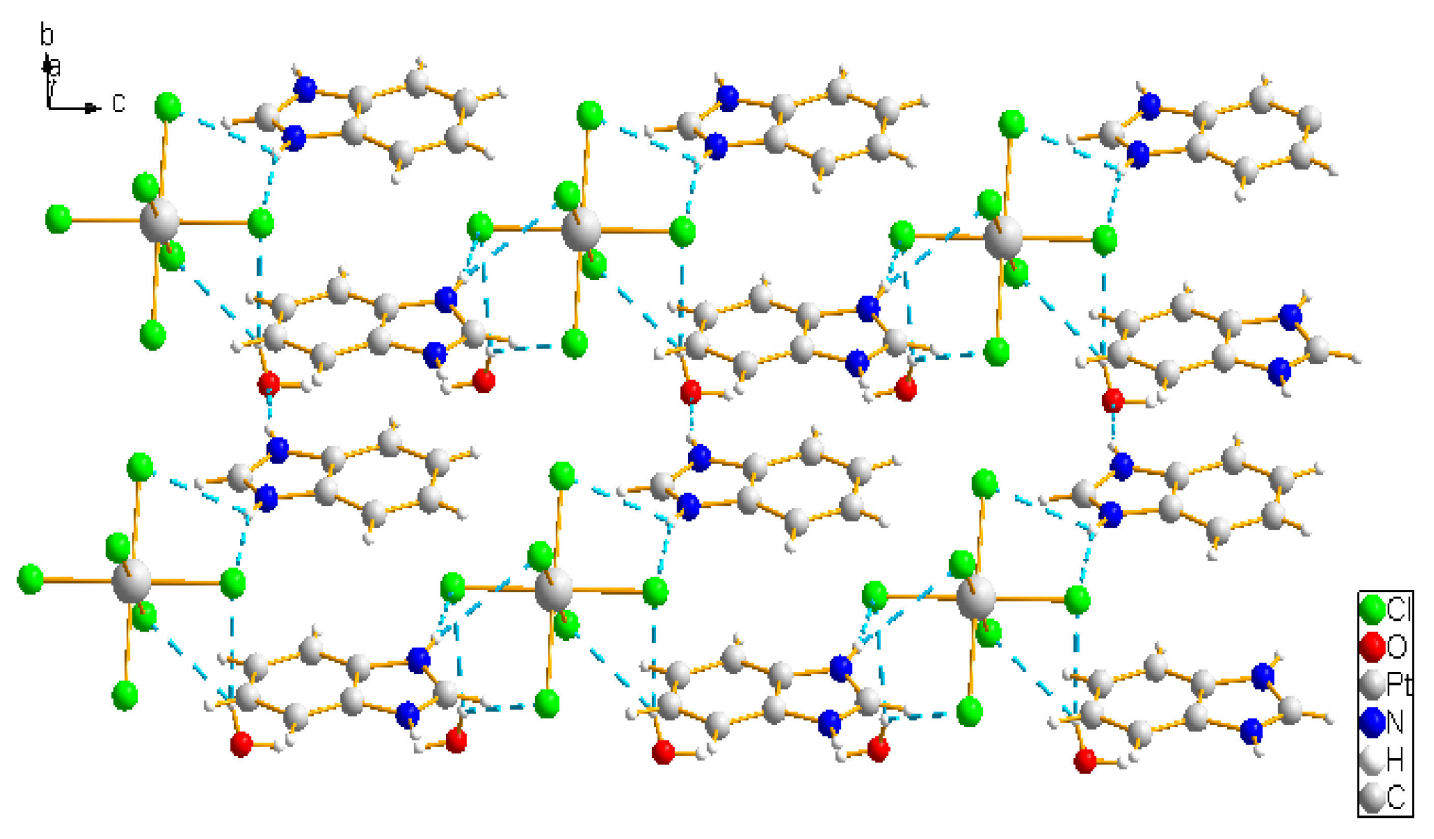
View of the asymmetric unit of complex **2** with the numbering scheme. The thermal probability is drawn at 30%. Symmetry code: i = −x, −y, −z.

**Figure 4:**
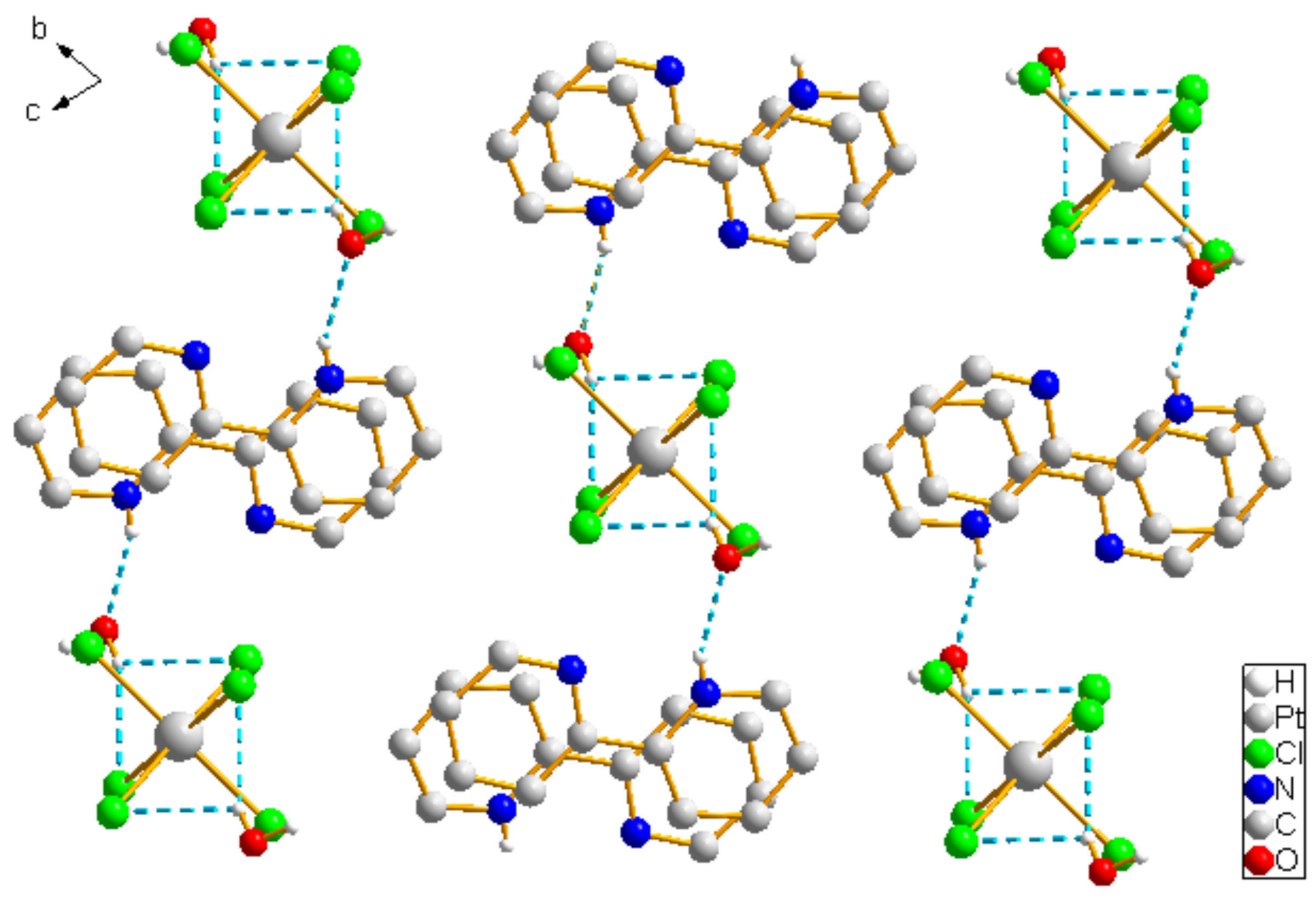
Hydrogen-bonded motifs and the packing graphic for complex **2**. Hydrogen bonds are shown as dashed lines.

Similar to complex **1**, complex **2** is also a cation–anion mode (Figure 3), consisting of [PtCl_6_]^2−^ and two [H-bipy]^+^ and two water molecules. The H atom bonding to the base N atom of bipy was transferred from 5-H_3_ssal. In **2**, the solvent water oxygen atom O1w acts as a donor bonding to the adjacent chloride ion, with the distance O1w–H1B···Cl1^i^ and O1w–H1B···Cl2^i^ [symmetry code: i, 1/2 + x, 1/2 + y, z] of 3.48(2) and 3.45(4) Å respectively. Additionally, also acting as an acceptor, O1w forms a hydrogen bond to the adjacent H atom of N^+^–H in [H-bipy]^+^ cation, with the distance N1–H1C···O1w of 2.718(9) Å, linking [PtCl_6_]^2−^ and to generate a 1D chain (Figure 4).

**Figure 5:**
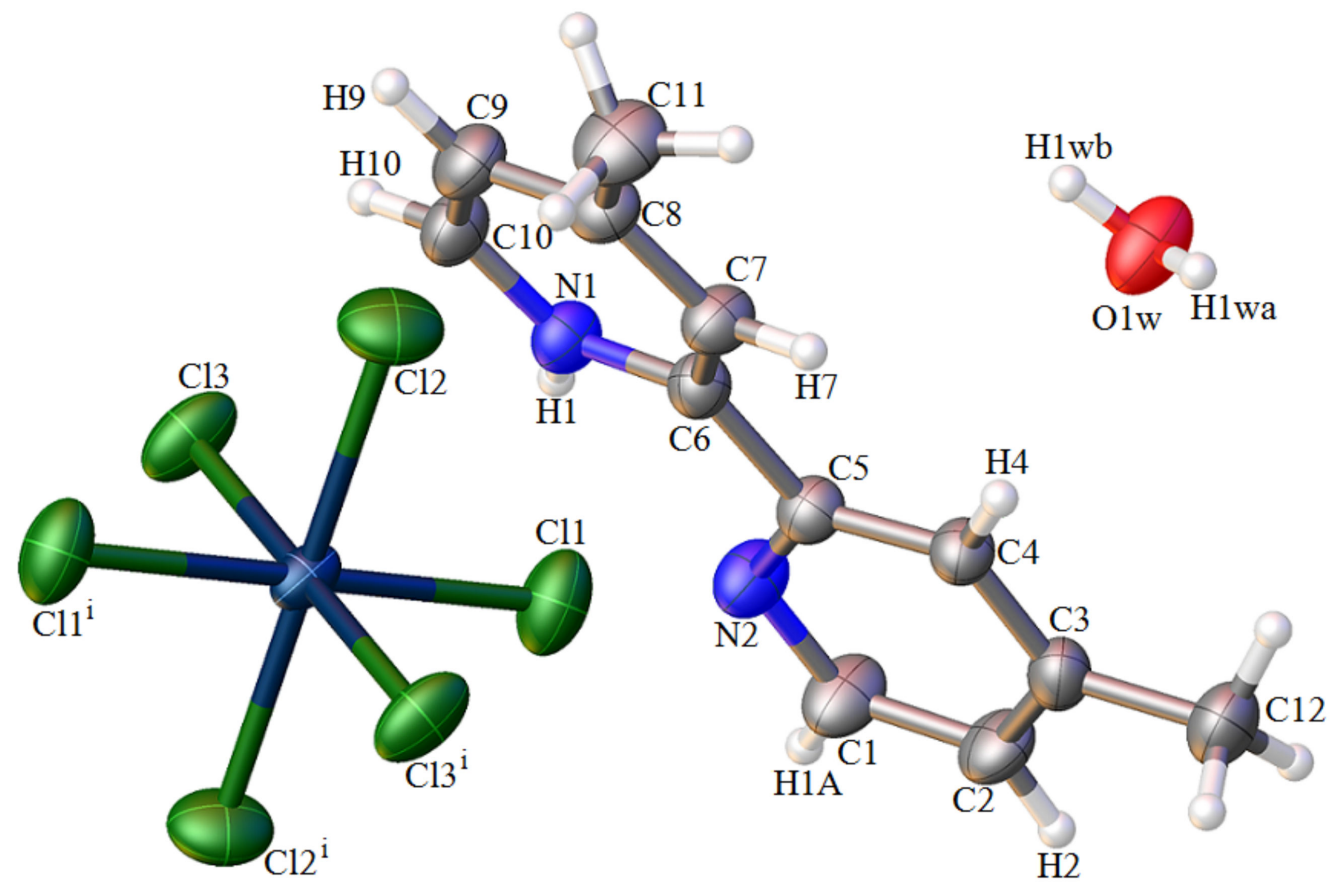
View of the asymmetric unit of complex **3** with the numbering scheme. The thermal probability is drawn at 30%. Symmetry code: i = 2 − x, −y, −z.

**Figure 6:**
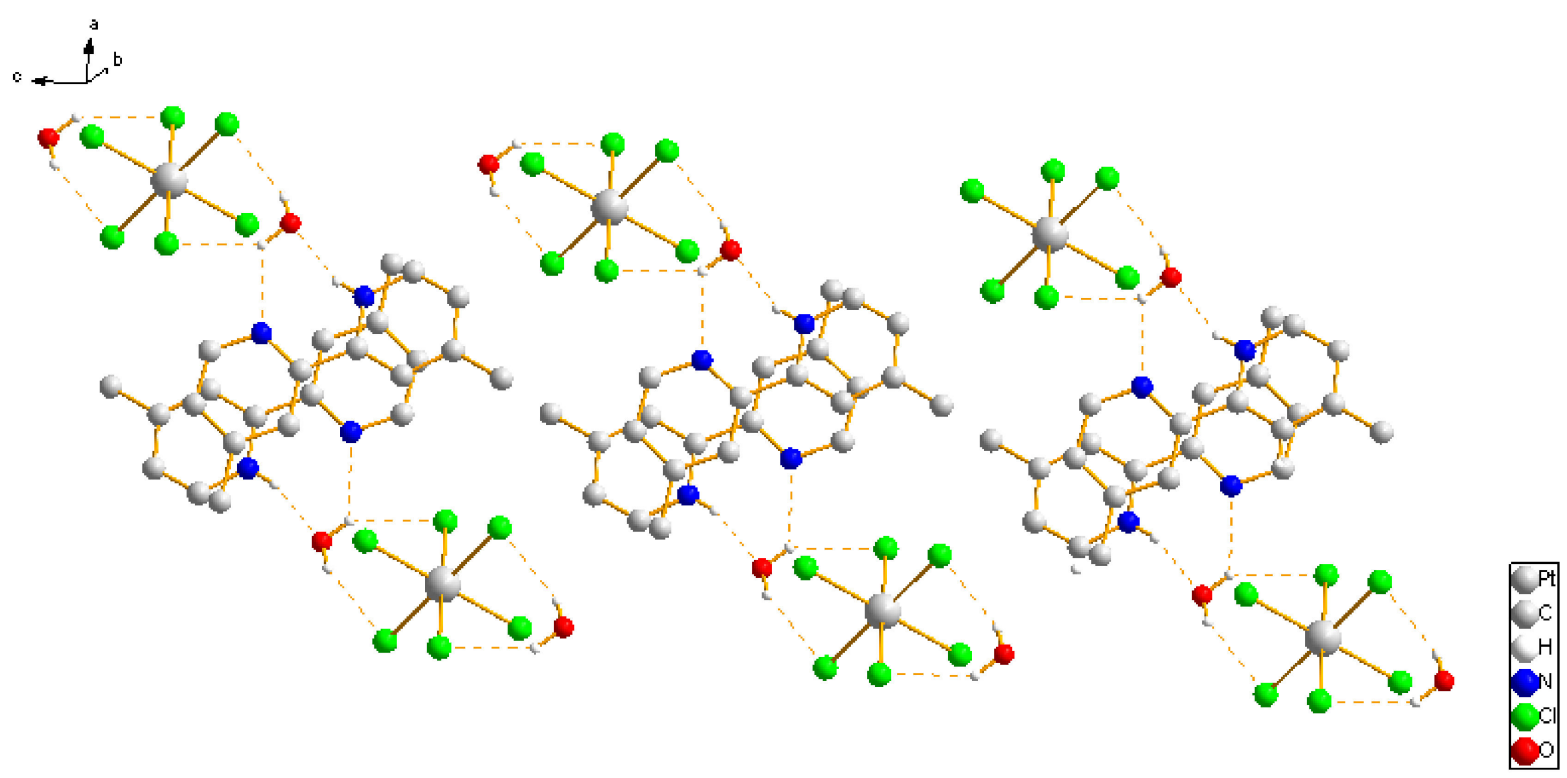
Hydrogen-bonded motifs and the packing graphic for complex **3**. Hydrogen bonds are shown as dashed lines.

Complex **3** is also a cation–anion species consisting of a [PtCl_6_]^2−^ cation, and two [H-dimethyl-bipy]^+^ anion, and two lattice water molecules (Figure 5). Hydrogen-bonding interactions, O1w–H1A···Cl1^i^, O1w–H1B···N2^ii^, O1w–H1B···Cl3^i^, N1–H1C···O1w^ii^ hydrogen bonds (Table 3), take important roles to build a 1D chain (Figure 6). From the above information on the three [PtCl_6_]^2^^−^ salt species, we discover that the primary hydrogen-bonding supramolecular synthons, such as N–H…O, O–H…Cl and N–H…Cl hydrogen bonds, are important and do matter in determining the intermolecular geometry. Moreover, the coulombic attractions between the anions and cations plays an important role in constructing these structures [28,29]. In addition, considering that the electrostatic potentials are not uniformly distributed on their surfaces and the more positive regions of the cations appear to want to align above or below the less positive regions, ion “stacking” is also helpful to construct these complexes [30]. The successful design of molecular salts therefore requires a careful balance of these various components of the lattice energy [31]. Desiraju-Wuest’s postulate that molecular synthons with appropriately “sticky” supramolecular functionalities can be used to design and prepare crystal structures is a useful, powerful, and attractive insight [32,33,34,35]. 

### 2.2. XRD and TG Analysis

The purities of these complexes were confirmed by XRD analysis, in which the experimental XRD patterns are consistent with those obtained from the stimulated XRDs based on the single crystals at room temperature (Figures S1–S3). The difference in reflection intensities between the simulated and experimental patterns is due to the variation in preferred orientation of the powder samples during the collection of the experimental XRD data. 

**Figure 7:**
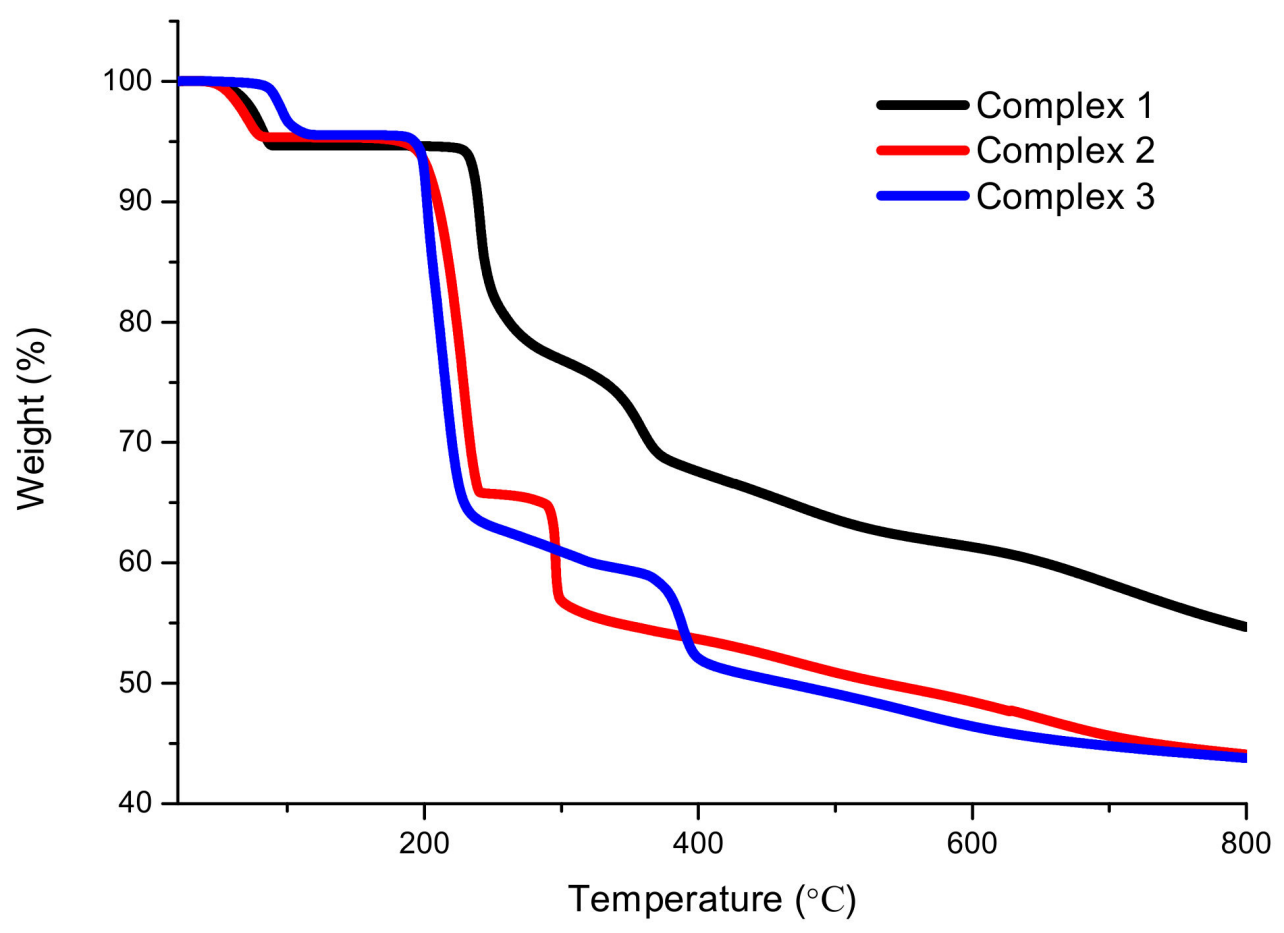
TGA curves of complexes **1**–**3**.

Thermogravimetric analysis (TGA) was conducted to study the stability of four complexes (Figure 7). All the thermal decomposition thermograms of the complexes show broad similarity. First, weight losses of these complexes occur from solvent molecules at low temperature, and then hydrogen bonds and electrostatic interactions disrupt the rise of temperature. 

Complex **1**–**2** similarly decomposed from 42 °C to 90 °C, with losses of 5.35% and 4.68%, respectively, which are attributed to the loss of solvent molecules (calculated at 5.30% for **1** and 4.75% for **2**). After a platform till to 225 °C, complex **1** lost 33.49% of its weight in the temperature range 225–425 °C, corresponding to the release of one of [H-bipy]^+^ (calculated 35.01%), and then the structure bankrupted smoothly. The second-step weight loss of 44.47% for complex **2** occurs from 185 °C to 319 °C, with a short platform between 245–281 °C, for the loss of two [H-bipy]^+^ (calculated at 41.17%). Different from **1**–**2**, complex **3** began to lose the two water molecules (experimental 5.18%, calculated 4.43%) between 90 °C and 120 °C, and it lost 47.89% of its weight from 185 °C to 405 °C, releasing two [H-dimethyl-bipy]^+^ (calculated at 45.58%). Then, without an obvious platform, the structure collapsed quickly.

### 2.3. Spectroscopic Analysis

**Figure 8:**
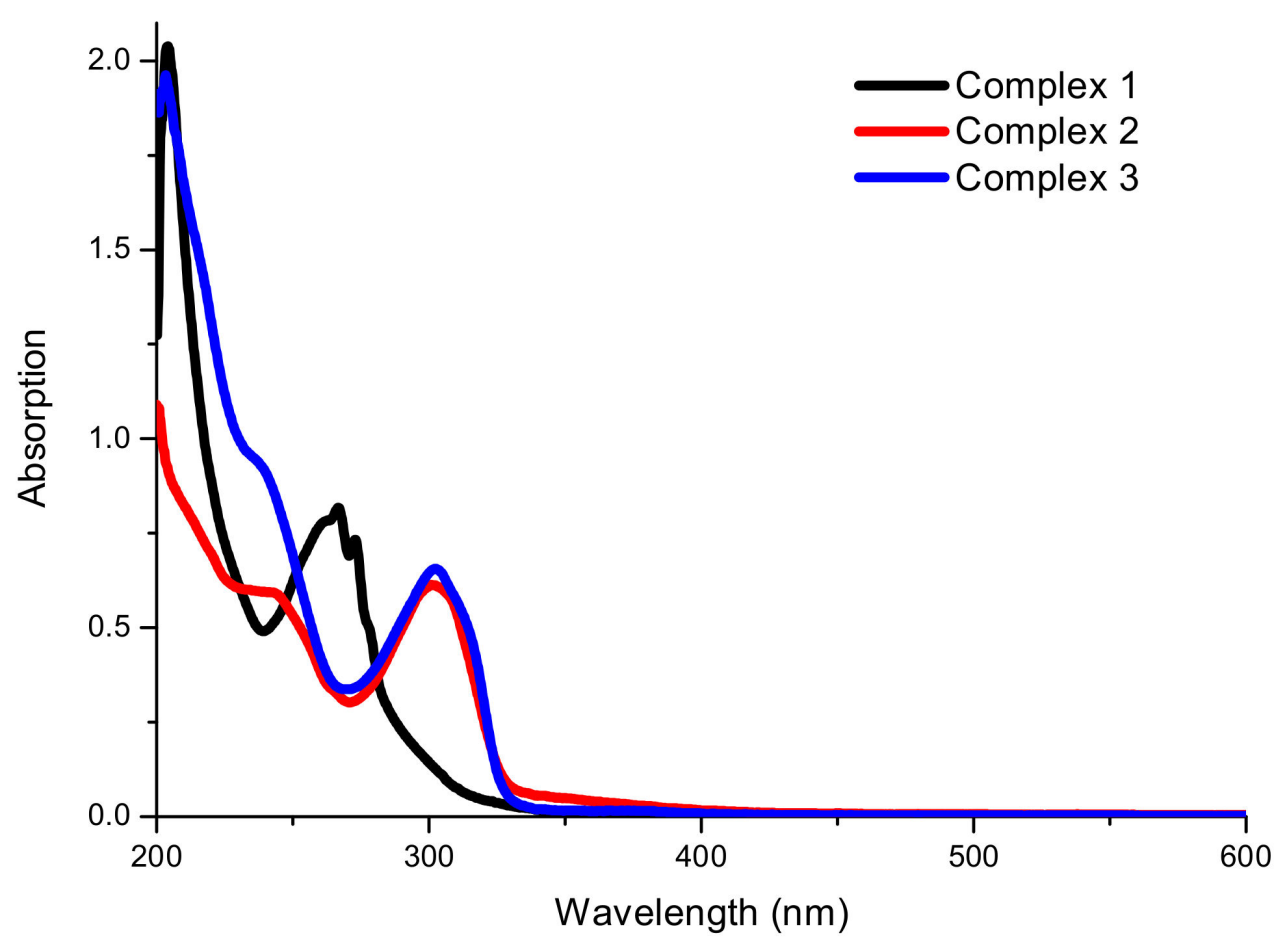
UV-vis spectra of complexes **1**–**3** in methanol.

In the infrared spectrum of these complexes (Figures S4–S6), intense bands can be observed around the 3493–3578 cm^−1^ and 3261–3058 cm^−1^ regions, assigned to the stretching modes of H_2_O and N–H. The intense bands between 1400 and 1600 cm^−1^ can be assigned to the coupled v(C=C)/v(C=N) stretching modes of the phenyl and pyridyl/imidazolyl rings, whereas another important and intense band appears at around 1000 cm^−1^ for pyridyl/imidazolyl rings. The strong bands at around 800 cm^−1^ belong to the coupled δ(C–N)/v(C–C) [36]. The UV-visible absorption spectra of **1**–**3** have been measured in methanol with a concentration of 2.0 × 10^−5^ mol/L (Figure 8). The UV/vis spectra display intense absorption bands ranging from 200 to 302 nm, indicating that electronic transitions are mostly π to π*, originating from the phenyl groups of ligands (Figure S7). The absorptions have more intensive absorptions than those of free ligands, confirming that the introduction of metal ions can enhance the absorptions (Figure 8 and Table 3). In addition, complexes **2**–**3** exhibit red-shift absorption, while complex **1** shows a little blue shift compared with bzm. 

**Figure 9:**
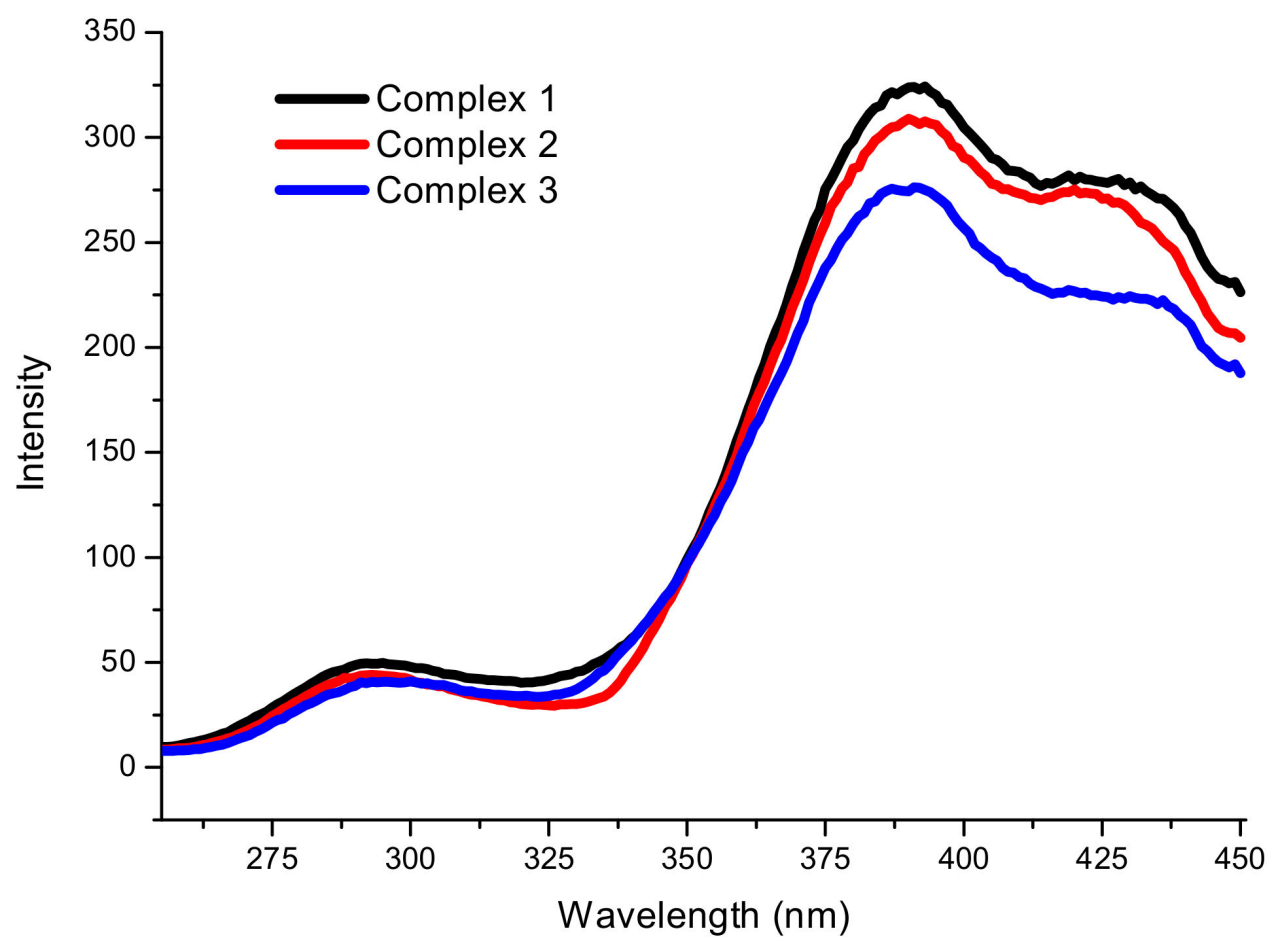
Solid-state emission spectra for complexes **1**–**3**.

The solid-state fluorescent properties of complexes **1**–**3** have been investigated at room temperature, as depicted in Figure 9. The maximum emissions for these complexes occur at 391 nm (λ_ex_ = 235 nm) with a broad shoulder at around 430 nm, which is similar to those of the free ligands (Figure S8), showing that the introduction of [PtCl_6_]^2−^ does not affect the fluorescent emissive position dramatically, while the strengths of the three complexes are all obviously lower than the corresponding ligands, and the emission of complex **1** is quenched completely at 300 nm, at which the bbmz ligand has a strong emission. The reason for the intensity reduction or even quenching probably is the introduction of metal ions and solvent molecules, which play an important role in weakening the fluorescent emission [37,38]. Meanwhile, the weak interactions, especially the hydrogen-bonding interactions, also play an important role in weakening the fluorescence intensity of the supermolecules [39,40,41]. 

### 2.4. In Vitro Antitumor Activities 

**Figure 10:**
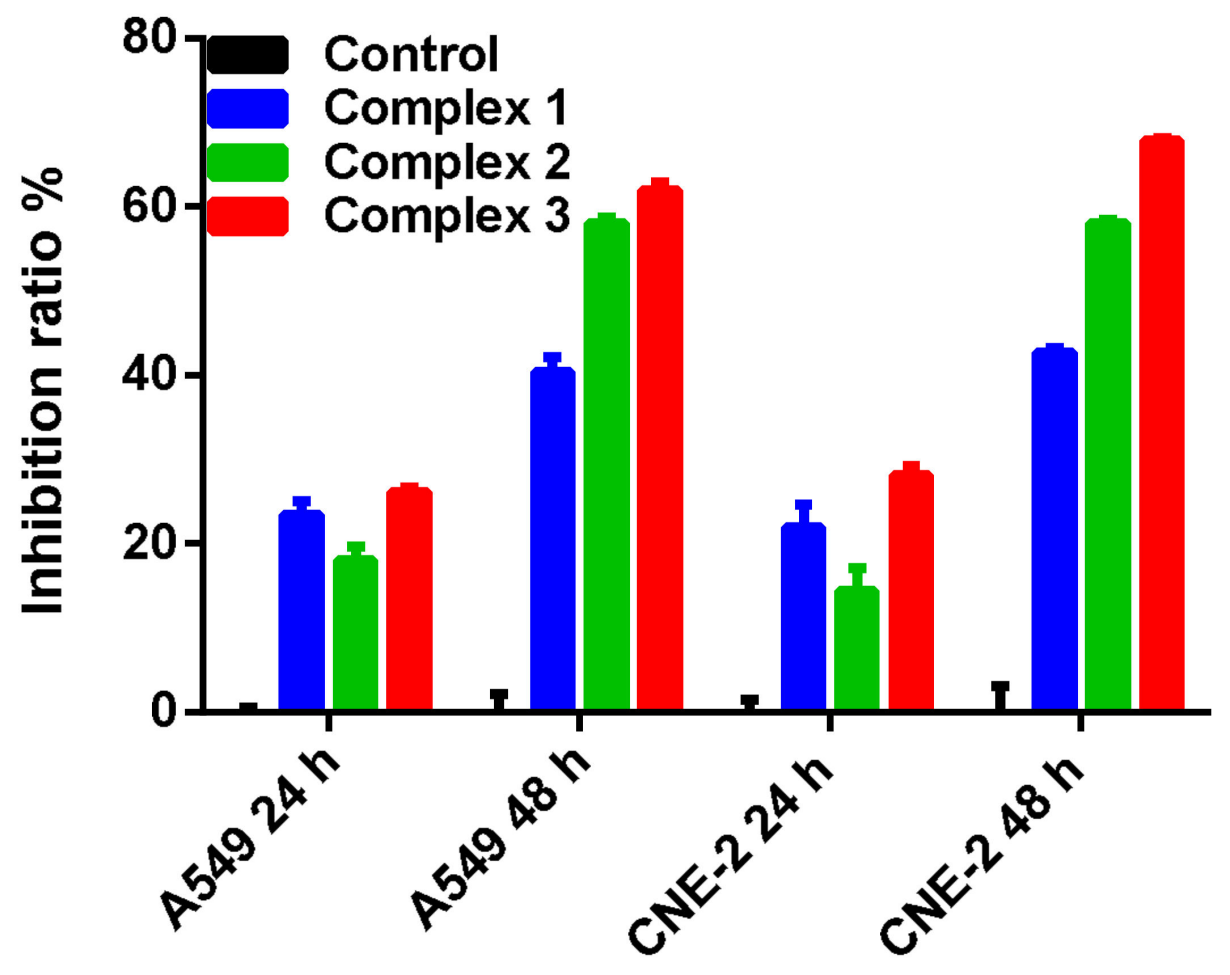
Induction of apoptosis in tumor cells by complexes **1**–**3**.

Nitrogen donor heterocyclic compounds such as imidazole and bipyridine have been found to possess a broad spectrum of biological activities such as anticancer, antitubercular, anti-inflammatory, analgesic, and antidepressant activities [42]. Combining bioactive compounds with a metal in a single molecule may result in a synergy that can translate into improved activity, and, based on this point, many new complexes participated by nitrogen heterocyclic ligands have been synthesized and characterized [43,44,45]. Here, the three complexes were examined for in vitro cytostatic activity against A549 cells and CNE-2 cells. As shown in Figure 10, we could conclude that complexes **1**–**3** showed the ability to promote apoptosis for the selected cell lines at both 24 and 48 h. For A549 cells at 24 h, 22.41%, 19.63%, and 24.22% inhibition ratio were observed respectively treated by complexes **1**–**3**, while 40.15%, 57.88%, and 62.26% at 48 h. For CNE-2 cells, 22.59%, 14.35%, and 28.12% were inhibited at 24 h respectively treated by complexes **1**–**3**, while 42.43%, 58.03%, and 67.88% at 48 h. The inhibition ratio of every compound increased obviously at 48 h with respect to that at 24 h, indicating that the inhibited proliferation is in a time-dependent manner, and complex **3** showed the best apoptotic activity among these complexes. 

**Figure 11:**
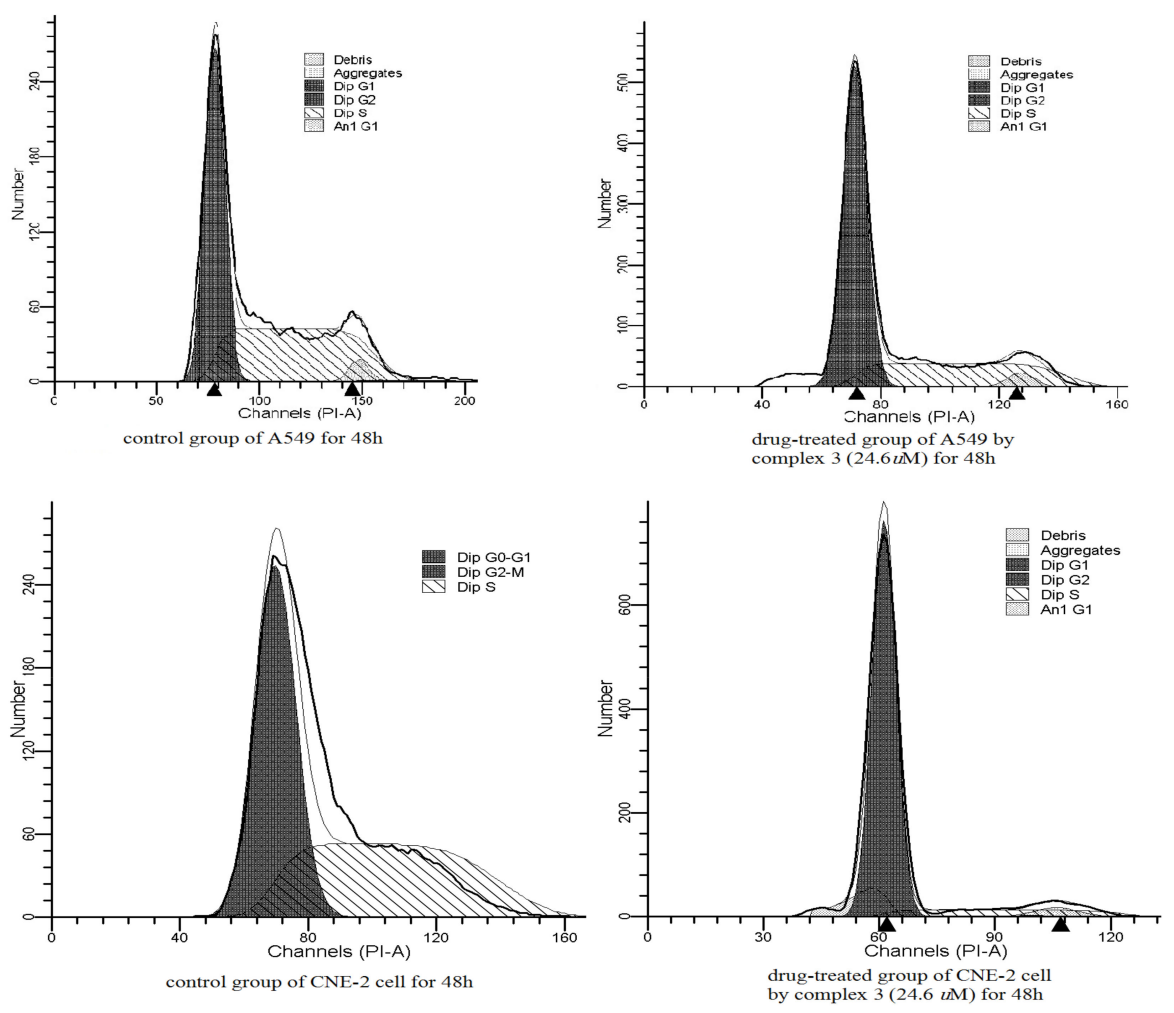
The cell cycles of A549 and CNE-2 treated with 24.6 μmol/L complex **3** for 48 h by FCM.

Further cell cycle for complex **3** was analyzed by flow cytometry (FCM), and results were expressed as a mean percentage of cells in different phases of the cell cycle (G_2_/M, S, G_0_/G_1_) and debris/necrosis cells. G_0_/G_1_ phase cells increased (16% for A549, and 28% for CNE-2), while the ratio of S and G_2_ phase cells was reduced accordingly (both about 17% for A549 and CNE-2), indicating that complex **3** arrested the cells in the G_0_/G_1_ phase (Figure 11). These results suggested that the agminated products of platinum salts and nitrogen heterocyclic compounds are still bioactive complexes and can induce apoptosis in cells. In addition, these compounds exhibited different antitumor abilities though they all contained the same platinum centers. Considering the difference of the ligands, the substituent groups on the nitrogen heterocyclic compounds may influence their biological activities, since CH_3_- in complex **3** is an electron-donating group, while C_6_H_5_- in complex **1** is an electron-withdrawing group.

## 3. Experimental Section

### 3.1. Materials and Physical Measurements

Chemicals were obtained from commercial sources with reagent grade. Elemental analyses for C, H, and N were carried out on a Perkin–Elmer analyzer model 1110 (Waltham, MA, USA). The infrared spectra were taken on a Nicolet Nexus 470 infrared spectrophotometer (Madison, WI, USA) as KBr pellets in the 450–4000 cm^−1^ region. The fluorescence spectra were measured using a SHIMADZU RF-540 spectrometer (Kyoto, Japan) on powdered samples in solid state at room temperature. Thermogravimetric analysis (TGA) was carried out on a Delta Series TA-SDT Q600 (New Castle, DE, USA) in a nitrogen atmosphere in the temperature range from room temperature to 800 °C (heating rate = 10 °C/min). UV-visible spectra for the complexes in CH_3_OH at room temperature were recorded on a Varian Bio 2550 UV-visible spectrophotometer (Santa Clara, CA, USA) in 1-cm quartz cuvettes in the range of 200–600 nm. XRD patterns were recorded on a Rigaku D/max-RA rotating anode X-ray diffractometer (Tokyo, Japan) with graphite-monochromatized Mo_K__a_ (λ = 0.71073 Å) radiation at room temperature. 

### 3.2. Synthesis of the Three Complexes 

[*PtCl*_6_]·(*H-bzm*)_2_·2(*H*_2_*O*) (**1**): KPtCl_6_ (0.048 g, 0.1 mmol) and 5-sulfosalicylic acid (5-H_3_ssal, 0.063 g, 0.25 mmol), were dissolved in 10 mL hot water. With heating and stirring, benzimidazole (0.037 g, 0.3 mmol) was added, and after stirring for 20 min, 5 mL acetonitrile was dropped into the above solution. After cooling to the room temperature, the clear solution was filtered and was set aside for evaporation. On the second day, dark yellow block-shaped crystals were collected by filtration. Yield: 65%. Anal. Calcd (%) for C14 H18 Cl6 N4 O2 Pt: C, 24.64; H, 2.64; N, 8.21. Found: C, 24.68; H, 2.48; N, 8.26. IR (KBr pellet, cm^−1^): 3578 (s), 3514 (s), 3261 (s), 2810 (s), 2730 (s), 1618 (m), 1597 (m), 1497 (m), 1445 (s), 1383 (s), 1265 (m), 1237 (s), 1136 (w), 1108 (m), 1003 (w), 971 (w), 865 (m), 756 (s), 702 (m), 621 (w), 598 (m), 542 (w), 512 (m), 416 (w).

[*PtCl*_6_]·(*H-bipy*)_2_·2(*H*_2_*O*) (**2**): The procedure was similar to the synthesis of **1**, except that benzimidazole was used instead of 2,2′-bipyridine, and red needle-shaped crystals were obtained. Yield: 65%. Anal. Calcd (%) for C20 H22 Cl6 N4 O2 Pt: C, 31.67; H, 2.90; N, 7.39. Found: C, 31.80; H, 2.89; N, 7.37. IR (KBr pellet, cm^−1^): 3557 (s), 3496 (s), 3153 (s), 3089 (s), 3062 (s), 1616 (s), 1603 (s), 1584 (s), 1530 (s), 1471 (m), 1429 (m), 1354 (w), 1319 (m), 1304 (m), 1277 (m), 1219 (m), 1149 (m), 1082 (m), 989 (m), 920 (w), 861 (w), 768 (s), 608 (w), 505 (w).

[*PtCl*_6_]·(*H-dimethyl-bipy*)_2_·2(*H*_2_*O*) (**3**): The procedure was similar to the synthesis of **1**, except that benzimidazole was used instead of 4,4′-dimethyl-2,2′-bipyridine. Yield: 57%. Anal. Calcd (%) for C24 H30 Cl6 N4 O2 Pt: C, 35.47; H, 3.69; N, 6.91. Found: C, 35.51; H, 3.67; N, 6.90. IR (KBr pellet, cm^−1^): 3493 (s), 3058 (s), 2924 (s), 1631 (s), 1598 (s), 1560 (m), 1521 (m), 1488 (w), 1451 (m), 1383 (w), 1357 (w), 1305 (m), 1280 (w), 1222 (m), 1120 (w), 992 (m), 950 (w), 825 (m), 507 (w).

### 3.3. X-ray Crystallography Measurement

Single crystals of complexes **1**–**3** suitable for X-ray diffraction analysis were used with an Oxford Diffraction Xcalibur CCD diffractometer using graphite monochromated Mo-Kα radiation (λ = 0.71073 Å) at room temperature. The frames were integrated with the CrysAlisPro package and the data were corrected for absorption using the program CrysAlisPro [46]. The structures were solved by direct methods using the program SHELXL-97 [47]. All non-hydrogen atoms were refined with anisotropic thermal parameters by full-matrix least-squares calculations on F^2^ using the program SHELXL-97. The hydrogen atoms attached to the phenyl group carbon atoms were put in calculated positions, and the coordinated water hydrogen atoms were located from the Fourier maps. The graphics were drawn by the ORTEP and Mercury [48,49]. Details of crystal data and structure refinements for the four complexes are listed in Table 1. CCDC1840975, 1840974, and 1840976 for complexes **1**–**3** contain the supplementary crystallographic data for this paper. These data can be obtained free of charge from the Cambridge Crystallographic Data Centre via www.ccdc.cam.ac.uk/data_request/cif.

### 3.4. In Vitro Antitumor Activity Assay 

The 3-(4,5-dimethylthiazol-2-yl)-2,5-diphenyltetrazoliumbromide (MTT) cell viability assay was performed. A549 and CNE-2 were seeded at a density of 6 × 10^3^ cells per well of a 96-well plate in 120 μL culture medium. Doxorubicin was used as a positive control, and PBS was used as a negative control. Two independent experiments, each run in triplicate, were performed. Each kind of cells was treated for 24 and 48 h with same concentration of the complexes. At the end of the treatment period, 25 μL of methylthiazoletetrazolium (MTT) solution (5 mg/mL PBS, pH 7.2) was added to each well. Following incubation for 4 h at 37 °C, solutions were carefully removed from each well and then 150 μL DMSO were added in each well to dissolve the MTT–formazan product. Absorption was measured at 490 nm on Synergy 2 Multi-Mode Microplate Reader (Bio Tek Instruments Inc., Winooski, VT, USA). Cell viability of treated cells was calculated in reference to the untreated control cells using the formula viability (%) = [100 × (sample Abs)/(control Abs)], where Abs is the absorbance value at 490 nm. 

### 3.5. Cell Cycle Analysis 

For cell cycle analysis, we also used A549 and CNE-2 grown in Dulbecco’s modified eagle medium (DMEM) supplemented with 5% (*v*/*v*) heat-inactivated fetal bovine serum and 2 mM l-glutamine at 37 °C in a humidified atmosphere of 5% CO_2_ and 95% air. Cells were periodically tested for Mycoplasma infection and found to be negative. Cells (3–5 × 10^5^ mL^−1^) untreated and drug-treated by complexes for 48 h were collected, washed three times with phosphate buffer saline (PBS), incubated for 1 h with 1 mg·mL^−1^ RNAse A and 20 μg·mL^−^^1^ propidium iodide at room temperature, and analyzed with a Becton Dickinson FACSCanto II flow cytometer (Franklin Lake, NJ, USA) to determine the percentage of cells in each phase of the cell cycle. 

## 4. Conclusions

Three complexes have been synthesized by hexachloroplatinate and nitrogen heterocyclic compounds in acidic conditions. They assembled through coulombic attractions and hydrogen bonding interactions. Moreover, ion “stacking” has also worked on the construction of these complexes. XRD spectra with powdered samples indicate consistency with the crystal structure. The antitumor activities of the complexes have been tested on two kinds of tumor cells and they all showed induction for the cell apoptosis for both A549 cells and CNE-2 cells, suggesting that the electron-donating substituent on the nitrogen heterocyclic rings may have a positive influence on the biological activity of active organic–metal complexes. 

## Figures and Tables

**Table 1:** Crystallographic data and refinement parameters for complexes **1**–**3**.

Complex	1	2	3
empirical	C14 H18 Cl6 N4 O2 Pt	C20 H22 Cl6 N4 O2 Pt	C24 H30 Cl6 N4 O2 Pt
fw	682.09	758.20	814.30
Cryst syst	Monoclinic	Triclinic	Triclinic
Space group	C 2/m	P −1	P −1
a (Å)	16.5752(11)	7.6476(5)	8.1295(4)
b (Å)	7.0641(5)	9.0089(6)	8.6453(4)
c (Å)	9.6557(7)	10.4610(7)	11.0288(5)
α (deg.)	90	69.925(1)	99.379(4)
β (deg.)	91.043(2)	73.123(1)	99.347(4)
γ (deg.)	90	80.402(2)	103.042(4)
*V* (Å^3^)	1130.39(14)	645.98(7)	728.86(6)
*Z*	2	1	1
D_c_ (g cm^−3^)	2.004	1.949	1.855
T (K)	293	293	293
μ (mm^−1^)	6.933	6.077	5.393
GOF	1.093	1.055	1.028
R1, wR2 (I > 2sigma)	0.018, 0.042	0.025, 0.074	0.032, 0.069
R1, wR2 (all data)	0.018, 0.042	0.027, 0.085	0.034, 0.070

**Table 2:** Geometries of the hydrogen bond lengths and bond angles in **1**–**3** (Å, °).

D–H···A	D–H	H···A	D···A	D–H···A
Complex **1**				
O1w–H1w ∙∙Cl1	0.813(18)	2.86(3)	3.551(3)	144(4)
O1w–H1w…Cl2 N1–H1 ∙∙O1w	0.813(18) 0.860(19)	2.84(3) 1.86(2)	3.5374(3) 2.720(5)	146(4) 177(6)
N2–H2 ∙∙Cl1	0.86	2.97	3.518(3)	123.7
N2–H2 ∙∙Cl2	0.86	2.50	3.316(4)	159.7
Complex **2**				
O1w–H1wb ∙∙Cl1^i^	0.851(10)	2.57(6)	3.381(6)	161(8)
N1–H1 ∙∙O1w^ii^	0.73(8)	2.11(8)	2.795(8)	157(8)
C10–H10 ∙∙O1w^iii^	0.93	2.66	3.295(10)	125.6
Complex **3**				
O1w–H1wa ∙∙Cl1^i^	0.85	2.51	3.295(3)	154.9
O1w–H1wb ∙∙N2^ii^	0.85	2.52	3.050(6)	121.3
O1w–H1B ∙∙Cl3^i^	0.85	2.60	3.267(4)	136.3
N1–H1 ∙∙O1w^ii^	0.82	2.01	2.794(5)	160.1

Symmetry codes: in **2**, i, x, y, 1 + z; ii, 1 − x, 1 − y, 1 − z; iii, x, y, −1 + z; in **3**, i, −1 + x, y, z; ii, 1 − x, 1 − y, 1 − z. O1w is the oxygen atom from lattice water molecules.

**Table 3:** UV-vis absorption spectral data for complexes **1**–**3** in methanol.

Complex	λ_max_/nm (ε/dm^3^ mol^−1^ cm^−1^)		
**1**	273 (3.77 × 10^4^)	266 (4.17 × 10^4^)	204 (1.04 × 10^5^)
**2**	302 (2.47 × 10^4^)	243 (2.41 × 10^4^)	201 (4.35 × 10^5^)
**3**	302 (2.56 × 10^4^)	237 (3.64 × 10^4^)	202 (7.60 × 10^4^)

## Supplementary Materials

The following are available online, Figures S1–S3: The simulative (bottom) and experimental (top) powder X-ray diffraction patterns for complex **1**–**3**, Figures S4–S6: The IR curve of complex **1**–**3**, Figure S7: UV-vis spectra of the organic ligands in methanol with the concentration of 2.0 × 10^−5^ mol/L, Figure S8: Solid-state emission spectra for these ligands (λ_ex_ = 300 nm).
