# Supplementary material for: Hydrogen-Bonded Organic–Inorganic Hybrid Based on Hexachloroplatinate and Nitrogen Heterocyclic Cations: Their Synthesis, Characterization, Crystal Structures, and Antitumor Activities In Vitro
Source: Molecules. 2018 Jun 8;23(6):1397. doi: 10.3390/molecules23061397 (PMC6099602; doi:10.3390/molecules23061397)
Supplement: Supplementary file 1 [file molecules-23-01397-s001.pdf]

Hydrogen-Bonded Organic-Inorganic Hybrid Salts Based on Hexachloroplatinate and Organic Cations: Synthesis, Characterization, Crystal Structures and their antitumor activity in vitro

Jin Zhao [†,1], Fuming Chen [†,1], Yutong Han [†,1], Huaqing Chen [2], Zhidong Luo [1], Hao Tian [1], Yi Zhao [1], Aiqing Ma [1,*] and Longguan Zhu [3,*]

[1] School of Pharmacy, Guangdong Medical University, Dongguan 523808, China; vincent-vg@163.com (J.Z.); fm.chen@siat.ac.cn (F.C.); hyt7570@163.com (Y.H.); luozhidong06@126.com (Z.L.); tianhao5588@126.com (H.T.); zhaoyicomnet@gdmu.edu.cn (Y.Z.)

[2] Guangdong Key Laboratory of Nanomedicine, CAS Key Lab for Health Informatics, Shenzhen Engineering Laboratory of Nanomedicine and Nanoformulation, Shenzhen Institutes of Advanced Technology (SIAT), Chinese Academy of Sciences, Shenzhen 518055, China; chenhuaqing99@163.com

[3] Department of Chemistry, Zhejiang University, Hangzhou 310027, China

* Correspondence: maqandght@126.com (A.M.); chezlg@zju.edu.cn (L.Z.); Tel.: +86-571-87951895

† These authors contribute equally to this work.

Supplementary Information

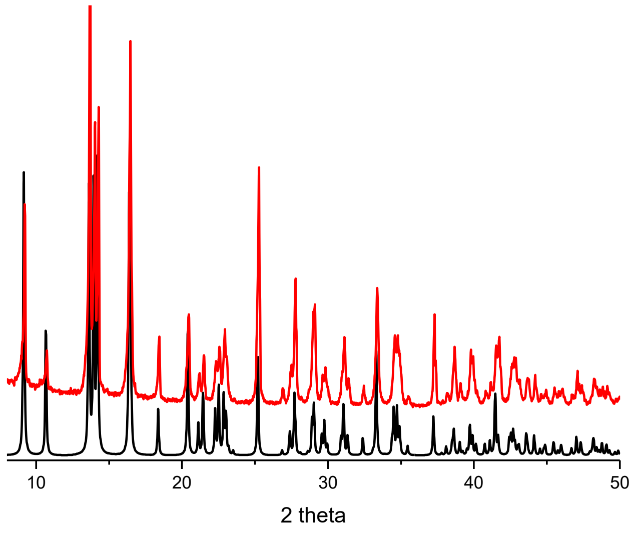

Figure S1. The simulative (bottom) and experimental (top) powder X-ray diffraction patterns for complex **1**.

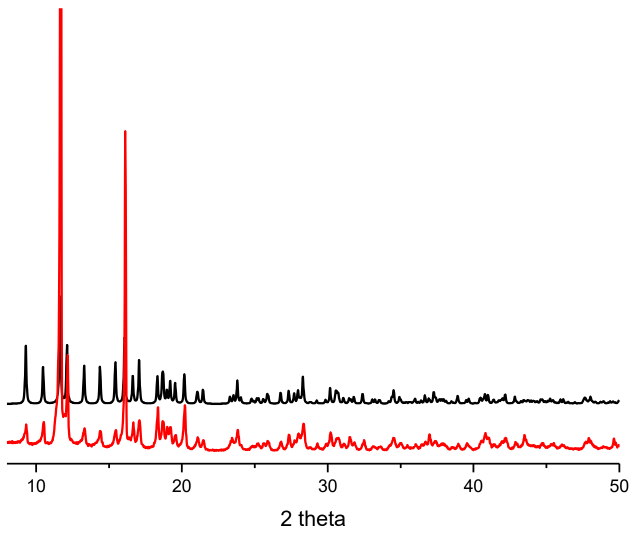

Figure S2. The simulative (bottom) and experimental (top) powder X-ray diffraction patterns for complex **2**.

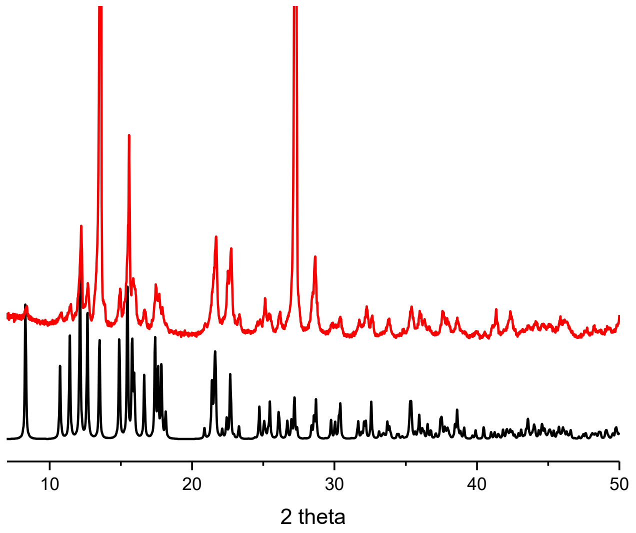

Figure S3. The simulative (bottom) and experimental (top) powder X-ray diffraction patterns for complex **3**.

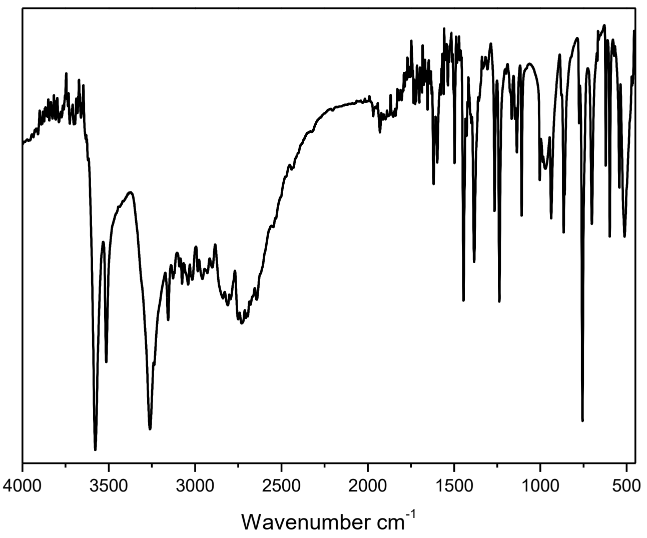

Figure S4. The IR curve of complex **1**.

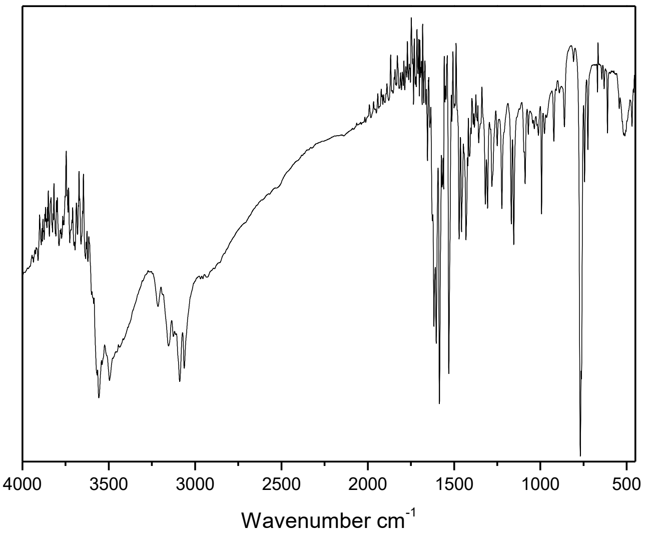

Figure S5. The IR curve of complex **2**.

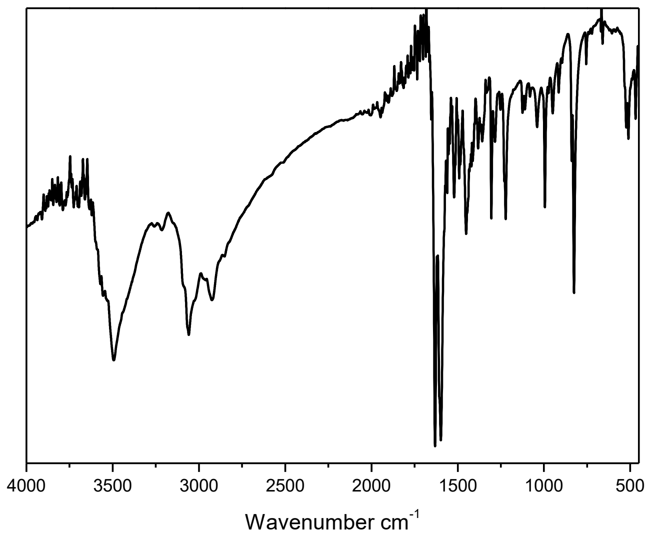

Figure S6. The IR curve of complex **3**.

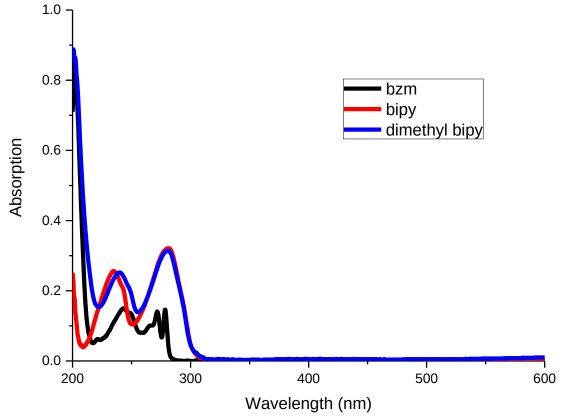

Figure S7. UV-vis spectra of the organic ligands in methanol with the concentration of 2.0 × 10^{-5} mol/L.

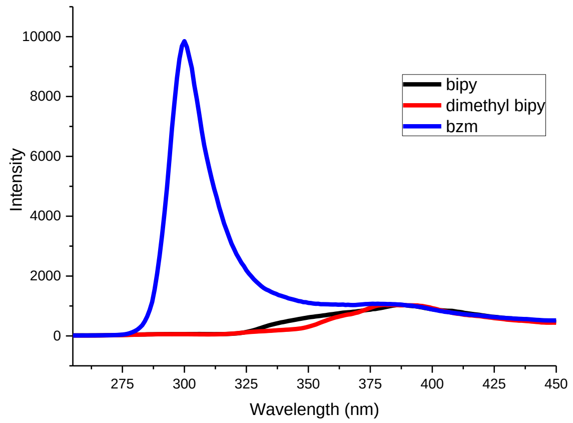

Figure S8. Solid-state emission spectra for these ligands (λ_{ex} = 300 nm)**.**
